# Expansion of the complex genotypic and phenotypic spectrum of *FGFR2*-associated neurocutaneous syndromes

**DOI:** 10.1007/s00439-023-02634-1

**Published:** 2024-01-24

**Authors:** Julia Schmidt, Silke Kaulfuß, Hagen Ott, Marianne Gaubert, Nadine Reintjes, Felix Bremmer, Steffi Dreha-Kulaczewski, Philipp Stroebel, Gökhan Yigit, Bernd Wollnik

**Affiliations:** 1 Institute of Human Genetics, University Medical Center Göttingen, Heinrich-Düker-Weg 12, 37073 Göttingen, Germany; 2 Department of Pediatric Dermatology, Children’s Hospital Auf Der Bult, Academic Hospital, Hannover, Germany; 3 Institute of Human Genetics, University Hospital Cologne, Cologne, Germany; 4 Institute of Pathology, University Medical Center Göttingen, Göttingen, Germany; 5 Department of Pediatrics and Adolescent Medicine, University Medical Center Göttingen, Göttingen, Germany; 6 DZHK (German Center for Cardiovascular Research), Partner Site Göttingen, Göttingen, Germany; 7 Cluster of Excellence “Multiscale Bioimaging: From Molecular Machines to Networks of Excitable Cells” (MBExC), University of Göttingen, Göttingen, Germany

## Abstract

The fibroblast growth factor receptors comprise a family of related but individually distinct tyrosine kinase receptors. Within this family, FGFR2 is a key regulator in many biological processes, e.g., cell proliferation, tumorigenesis, metastasis, and angiogenesis. Heterozygous activating non-mosaic germline variants in *FGFR2* have been linked to numerous autosomal dominantly inherited disorders including several craniosynostoses and skeletal dysplasia syndromes. We report on a girl with cutaneous nevi, ocular malformations, macrocephaly, mild developmental delay, and the initial clinical diagnosis of Schimmelpenning–Feuerstein–Mims syndrome, a very rare mosaic neurocutaneous disorder caused by postzygotic missense variants in *HRAS*, *KRAS,* and *NRAS*. Exome sequencing of blood and affected skin tissue identified the mosaic variant c.1647=/T > G p.(Asn549=/Lys) in FGFR2, upstream of the RAS signaling pathway. The variant is located in the tyrosine kinase domain of FGFR2 in a region that regulates the activity of the receptor and structural mapping and functional characterization revealed that it results in constitutive receptor activation. Overall, our findings indicate *FGFR2*-associated neurocutaneous syndrome as the accurate clinical-molecular diagnosis for the reported individual, and thereby expand the complex genotypic and phenotypic spectrum of *FGFR*-associated disorders. We conclude that molecular analysis of *FGFR2* should be considered in the genetic workup of individuals with the clinical suspicion of a mosaic neurocutaneous condition, as the knowledge of the molecular cause might have relevant implications for genetic counseling, prognosis, tumor surveillance and potential treatment options.

## Introduction

The fibroblast growth factor receptor (FGFR) 2 (MIM 176943, NM_000141.5) belongs to a family of related but individually distinct tyrosine kinase receptors. The FGFRs share a similar structure containing an extracellular ligand binding domain composed of three immunoglobulin-like domains, a single transmembrane helix segment, and an intracellular tyrosine kinase domain (Dionne et al. 1990; Houssaint et al. 1990; Gilbert et al. 1993; Pellegrini et al. 2000). These receptors are expressed on numerous cell types in various tissues and regulate crucial biological processes, including cell proliferation, migration, survival, and differentiation by activation of downstream signaling pathways, such as the PI3K-AKT-mTOR, PLCγ-PKC, or the RAS-MAPK signaling cascades (Kouhara et al. 1997; Thisse and Thisse 2005; Schubbert et al. 2007; Turner and Grose 2010). Pathogenic *FGFR* variants affecting ligand binding and specificity or tyrosine kinase activity can lead to aberrant receptor signaling causing diverse inherited conditions, e.g., skeletal disorders such as Pfeiffer syndrome (MIM 101600), Muenke syndrome (MIM 602849), or achondroplasia (MIM 100800). Activating germline variants in *FGFR2* have been linked to several autosomal dominantly inherited congenital malformation syndromes such as Apert syndrome (MIM 101200), Crouzon syndrome (MIM 123500), Jackson–Weiss syndrome (MIM 123150), and Pfeiffer syndrome (MIM 101600). Furthermore, aberrant cell growth due to FGFR alteration is known to play an important role in tumorigenesis (Chesi et al. 1997; Cappellen et al. 1999; Blume-Jensen and Hunter 2001; van Rhijn et al. 2002). In line with this, postzygotic somatic activating *FGFR2* alterations were found in various cancer types including colon, breast, gastric, endometrial, esophageal and cholangiocarcinoma (Kunii et al. 2008; Zhang et al. 2009; Reintjes et al. 2013; Mathur et al. 2014; Kwak et al. 2015; Helsten et al. 2016; Smyth et al. 2017; Shi et al. 2018). Interestingly, mosaic activating variants in *FGFR1* have been reported to cause encephalocraniocutaneous lipomatosis (ECCL; MIM 613001), a neurocutaneous condition characterized by ocular anomalies, skin lesions, and central nervous system anomalies (Moog 2009; Bennett et al. 2016). To identify the causative postzygotic variants in those ultra-rare disorders is often difficult, as they may not be detectable in genomic DNA from standard peripheral blood samples, but only in affected/dysregulated tissue. Therefore, a specific clinical suspicion and selection of the appropriate tissue for genetic analysis might be key to pinpoint these mosaic conditions. In general, mosaicism should be suspected when asymmetric disproportionate growth or typical cutaneous manifestations are present, e.g., hyperpigmentation following Blaschko lines or cerebriform connective tissue nevi.

Here, we report on an individual with cutaneous nevi (widespread nevus sebaceous on the face, skin pigmentation restricted to the right side of her neck, and epidermal nevi along the Blaschko lines), ocular malformations, macrocephaly, and mild developmental delay in whom we identified the mosaic variant c.1647=/T > G p.(Asn549=/Lys) in *FGFR2*. The variant is located in the tyrosine kinase domain of FGFR2 in a region that regulates the activity of the receptor. Protein structure simulation showed that the identified variant most likely results in constitutive receptor activation. Further functional characterization of the tyrosine kinase activity revealed a strongly elevated FGFR2 phosphorylation level, indicating an activating nature of this variant, which most likely induces an increased activation of downstream effectors of the FGFR2 pathway. Together, our findings expand the complex genotypic and phenotypic spectrum of *FGFR2*-associated syndromes. We conclude that the molecular analysis of *FGFR2* should be included in the genetic workup of individuals with the clinical suspicion of a mosaic neurocutaneous condition.

## Results

### Clinical report

**Fig. 1:**
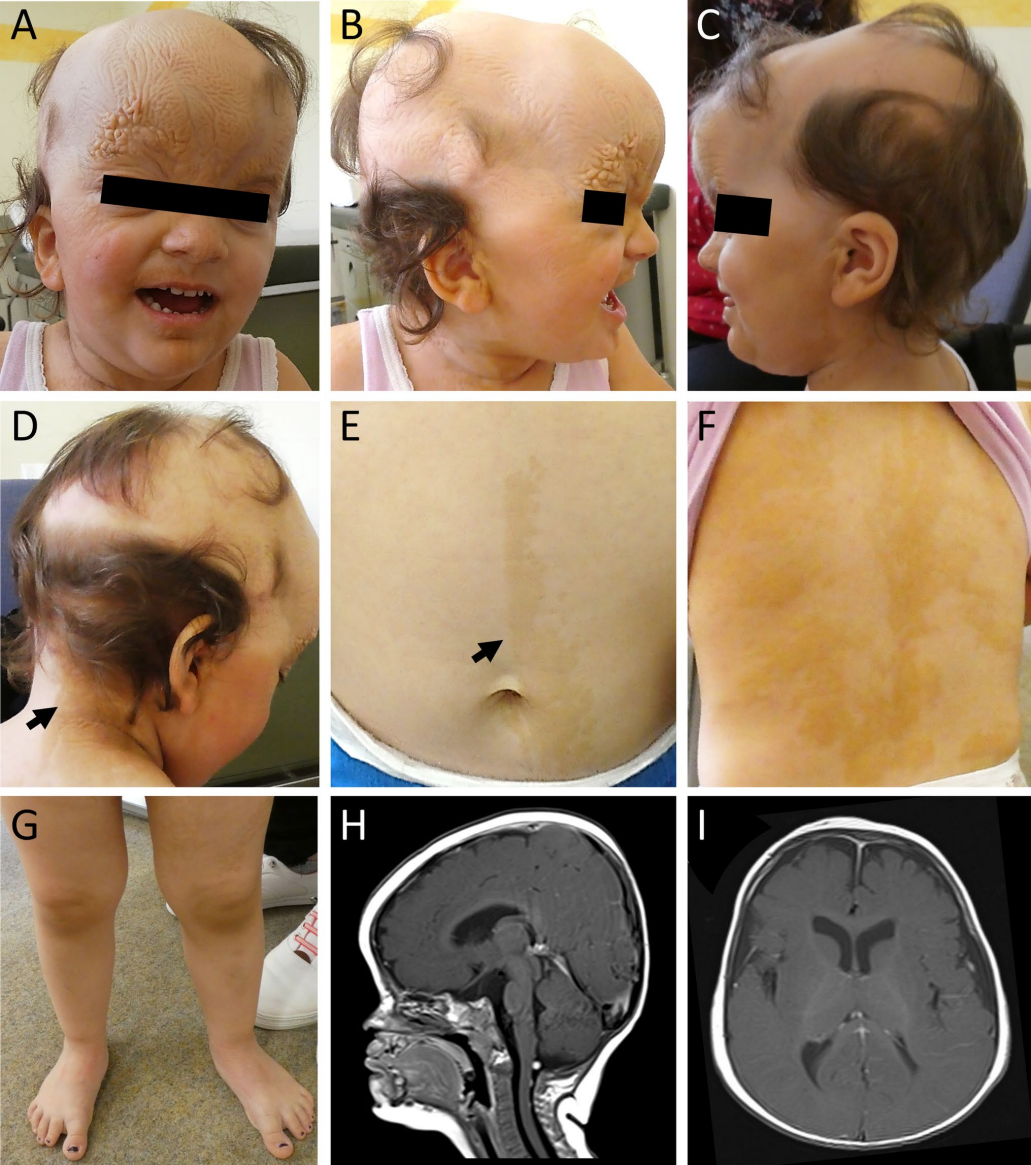
Clinical pictures and cranial images of the affected individual. **A**–**C** Clinical characteristics of the affected girl at the age of 2 years and 9 months included widespread nevus sebaceous on the face affecting the right eyebrow and areas of hair loss on the scalp, hypertelorism and low-set ears. **D** Hairless areas on the head and patchy skin pigmentation restricted to the right side of her neck (indicated by black arrow). **E** Linear epidermal nevi restricted to the left side of the abdomen (indicated by black arrow). **F** Widespread epidermal nevi on the torso. **G** Broad great toes on both sides. **H** Cranial T1 weighted mid-sagittal MRI (with gadolinium) at the age of 6 months depicting abnormal shape of the skull and a hypoplastic midface. Skull base appears dysmorphic with a steep clivus, a shallow sella and an enlarged posterior fossa. **I** Cranial T1 weighted axial MRI shows slightly widened internal cerebrospinal fluid spaces

A girl aged 2 years and 9 months with cutaneous nevi, malformations of the eye (defects of the retina/choroid, corneal clouding, strabismus), macrocephaly, and mild developmental delay (Fig. 1A–I) was referred to our Institute of Human Genetics for clinical and diagnostic evaluation. The girl was born to unrelated healthy parents at 39 + 2 weeks of gestation. Birth weight was 3965 g (+ 0.8 SD), length 52.2 cm (+ 0.6 SD), and OFC 38 cm (+ 2.5 SD). Pregnancy and family history were unremarkable. She presented with segmental, skin-colored, slightly elevated plaques over scalp, midface, chest, and upper limbs. In addition, hypertelorism, a hypoplastic midface, low-set ears, strabismus, corneal clouding, defects of the retina and choroid as well as broad great toes on both sides were observed. Clinical examination at the age of 2 years and 9 months revealed a height of 91 cm (-0.6 SD), a weight of 14.6 kg (+ 0.5 SD), and a head circumference of 53 cm (+ 2.8 SD). Her development was slightly delayed. Earlier genetic testing including chromosomal analysis, array CGH, and exome sequencing performed on genomic DNA extracted from blood were unremarkable. Histology of a biopsy from the skin lesion in the neck revealed hyperkeratosis, acanthosis, and papillomatosis. The findings were consistent with nevus sebaceous, the dermatologic hallmark of Schimmelpenning–Feuerstein–Mims syndrome (SFM; MIM 163200). Cranial MRI at the age of 6 months revealed an abnormal shape of the skull, hypoplastic midface, slightly pronounced intracerebral cerebrospinal fluid spaces and a dysmorphic skull base with a steep clivus, a shallow sella and an enlarged posterior fossa (Fig. 1H, I). The shape of the skull might point towards partial synostosis, but there were no computed tomography scans or radiographies of the skull to clearly depict a craniosynostosis. Investigations of bone metabolic parameters showed no abnormalities.

### Genetic analysis

**Fig. 2:**
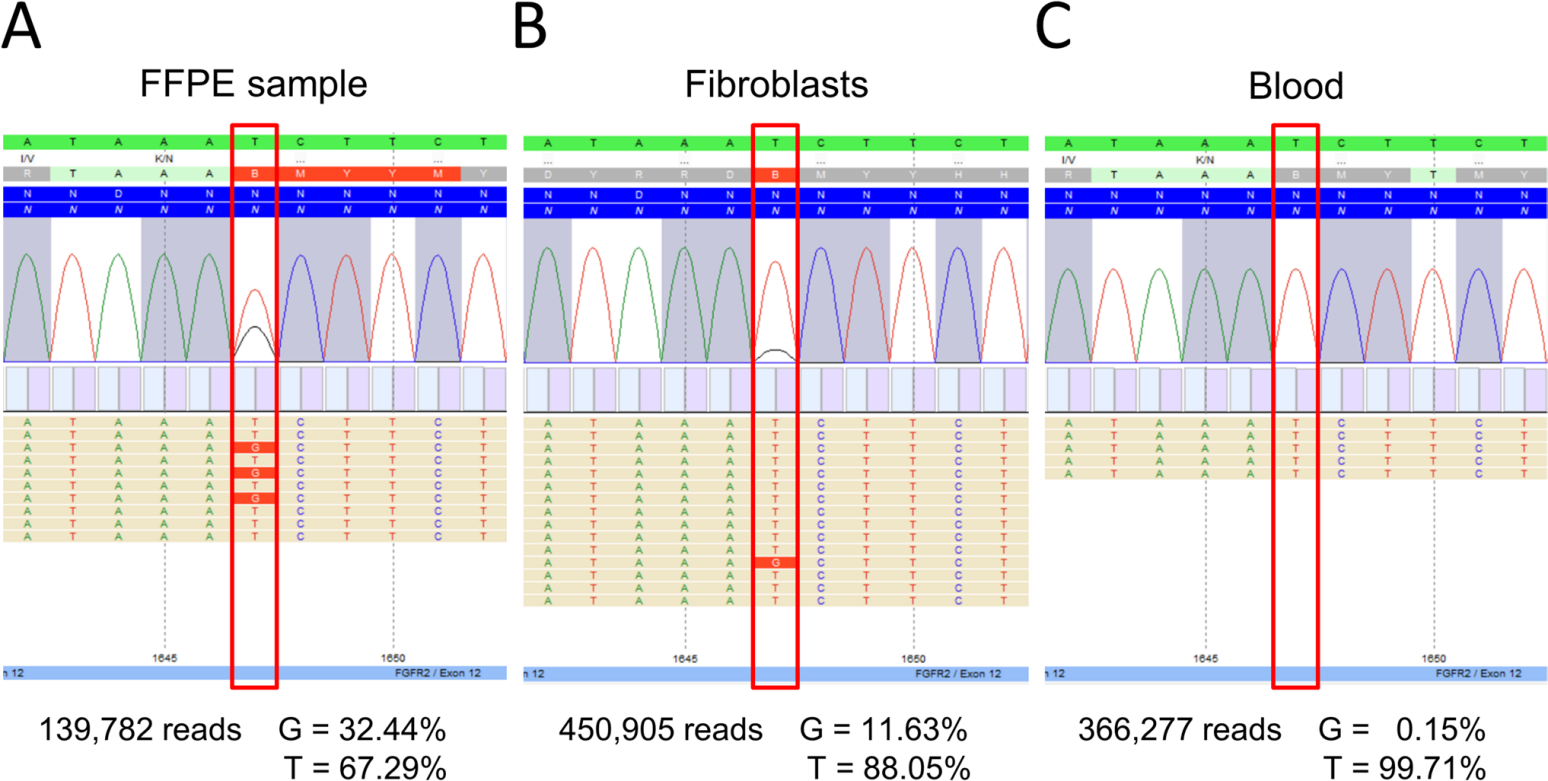
Molecular findings. Deep sequencing analysis was used to validate the exome data and confirmed the mosaic *FGFR2* variant c.1647=/T > G p.(Asn549=/Lys) in DNA extracted from biopsy tissue. The variant was present in 32% (45,352/139,782) of the reads from the FFPE sample (**A**), in 12% (52,451/450,905) of the fibroblasts (**B**) and was not (only 0.15%) detectable in DNA extracted from peripheral blood lymphocytes (545/366,227) (**C**)

Written informed consent was obtained from the patient’s parents prior to the participation in the study. The study was approved by the Ethics Committee of University Medical Center Göttingen (approval number 3/2/16) and performed in accordance with the Declaration of Helsinki protocols. DNA isolation from EDTA blood was carried out following standard protocols. Based on the initial tentative diagnosis of SFM syndrome, we organized a skin biopsy of an affected area in the neck, extracted genomic DNA from fibroblasts by standard extraction procedures, and performed a high-coverage custom-designed multigene panel including 116 known disease-associated genes including *HRAS*, *KRAS*, and *NRAS*, known to be linked to SFM syndrome (mean read depth of 4668 reads)*.* Despite intense analysis, we did not identify any likely causative variant. It is known that causative variants linked to SFM syndrome typically are detectable in the keratinocytes of the affected skin regions, but not in the fibroblasts underlying the altered epithelia (Friedrich et al. 2022). To exclude that the percentage of affected cells in the biopsy might have been too low to detect the causative variant, we decided next to extract genomic DNA from a FFPE skin sample (mainly including keratinocytes of the affected lesion) and repeated the high-coverage custom-designed multigene panel including *HRAS, KRAS,* and *NRAS* (mean read depth of 6465 reads)*.* Still, we were not able to identify a likely causative variant. Therefore, we proceeded by performing exome sequencing (ES) on DNA extracted from peripheral blood and DNA extracted from the skin biopsy, using Agilent SureSelect Human All Exon V7 (Agilent Technologies, Santa Clara, CA) and the Illumina NextSeq sequencer (Illumina, San Diego, CA). ES data were analyzed using Varvis 1.20.1 (Limbus Medical Technologies GmbH, Rostock, Germany) based on gnomAD Exomes + Genomes 2.1.1, dbSNP 142 and ClinVar 202,107. After in-depth filtering and variant interpretation, we identified a mosaic *FGFR2* variant NM_000141.5:c.1647=/T > G p.(Asn549=/Lys) in the nevus sample. Deep sequencing analysis was used to validate the exome data and confirmed the mosaic variant in DNA extracted from biopsy tissue: the variant was present in 32% (45,352/139,782) of the reads from the FFPE sample, in 12% (52,451/450,905) of the fibroblasts and was not (only in 0.15%) detectable in DNA extracted from peripheral blood lymphocytes (545/366,227) (Fig. 2A–C), suggesting that the variant was postzygotic. The variant, c.1647T > G, is located in exon 12 of the *FGFR2* gene and leads to the substitution of an asparagine at the amino acid position 549 by lysine (p.(Asn549Lys)). The variant is predicted as disease-causing by MutationTaster (http://www.mutationtaster.org), damaging by FATHMM (http://fathmm.biocompute.org.uk), and damaging by M-CAP (http://bejerano.stanford.edu/MCAP/). This variant has been reported as a somatic likely pathogenic variant in an endometrial endometrioid adenocarcinoma (ClinVar Variation ID: 376156; (Dutt et al. 2008)). The variant was not present in any current database of human genetic variations including the gnomAD database (https://gnomad.broadinstitute.org; last access date 22/02/2023).

### Structural mapping

**Fig. 3:**
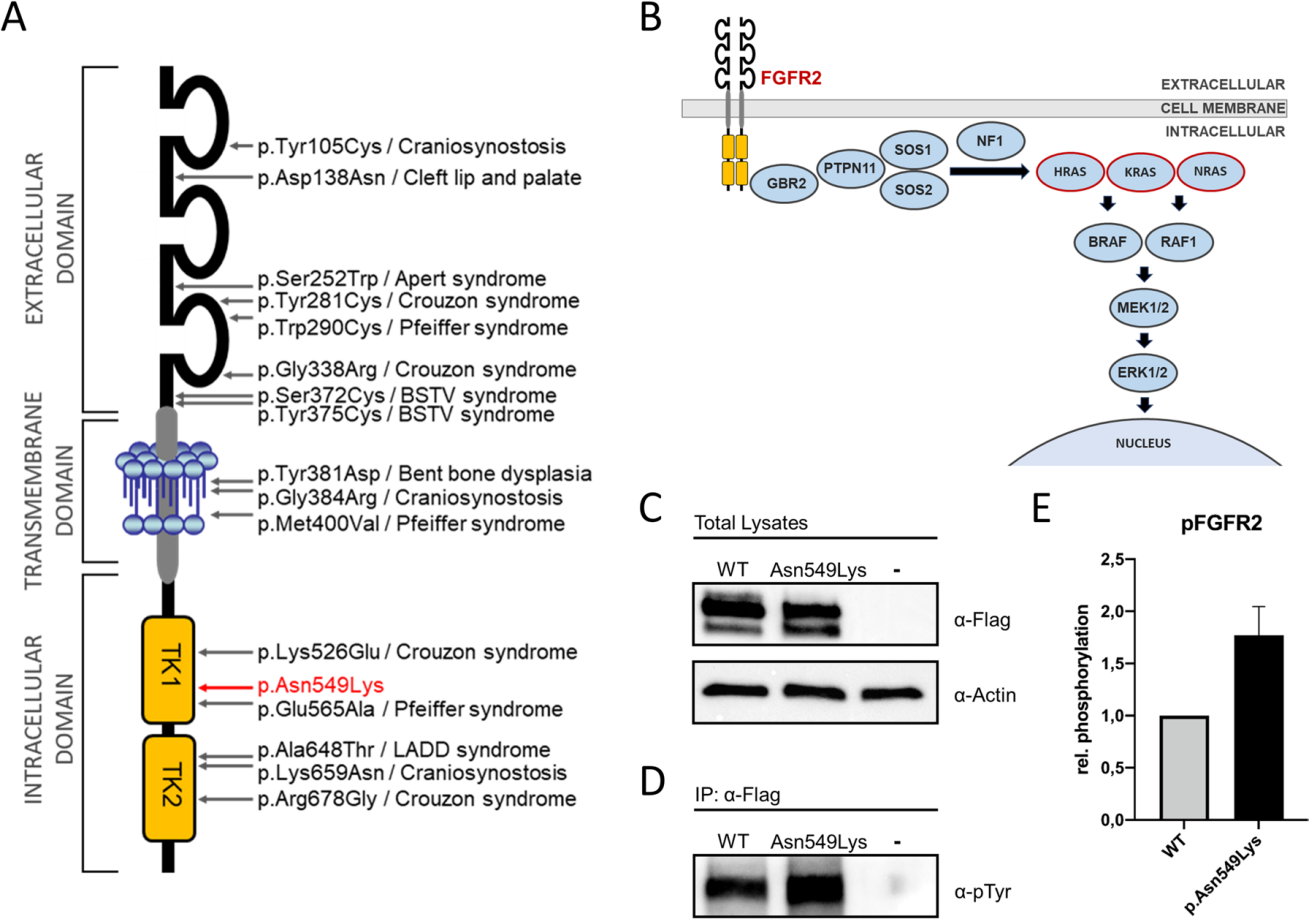
Overview of the RAS-MAPK signaling pathway, location and functional characterization of the p.(Asn549Lys) variant in FGFR2. **A** Schematic overview of the FGFR2 receptor. Red arrow points to the location of the variant identified in this study. TK1, tyrosine kinase domain 1; TK2, tyrosine kinase domain 2. Selected previously reported variants causing different *FGFR2*-associated disorders and their location are indicated by grey arrows. BSTV syndrome, Beare–Stevenson cutis gyrata syndrome. LADD syndrome, lacrimoauriculodentodigital syndrome. **B** Postzygotic activating variants in HRAS, KRAS and NRAS (indicated in red) represent known causes of SFM syndrome. The identified mosaic variant reported here leads to a constitutive activation of fibroblast growth factor receptor 2 (FGFR2) upstream of the RAS signaling cascade. **C** HEK293 cells were transiently transfected with vectors containing cDNA for FLAG-tagged wild-type *FGFR2* (WT) or *FGFR2* harboring the identified variant c.1647T > G p.(Asn549Lys). Untransfected cells served as negative control (−). Western blot analysis of FGRF2 expression revealed equal protein expression of wild-type and p.(Asn549Lys) FGFR2 (upper panel). Equal protein loading was confirmed by re-probing of the membrane with antibodies against actin (lower panel). **D** Representative Western blot showing increased tyrosine kinase activity of FGFR2 carrying the p.(Asn549Lys) amino acid substitution compared to wild-type FGFR2. HEK293 cells were transiently transfected with FLAG-tagged wild-type FGFR2, FGFR2 p.(Asn549Lys), or left untransfected as control (–). Lysates from cells were subjected to immunoprecipitation (IP) with anti-FLAG antibodies followed by SDS-PAGE and immunoblotting with anti-p-Tyr antibodies. **E** Quantification of pFGFR2 levels in cells expressing wild-type FGRF2 (WT) or FGFR2 harboring the identified amino acid substitution p.(Asn549Lys). pFGFR2 signal was significantly higher in cells expressing the p.(Asn549Lys) FGFR2 variant (*n* = 3; mean ± SD)

**Fig. 4:**
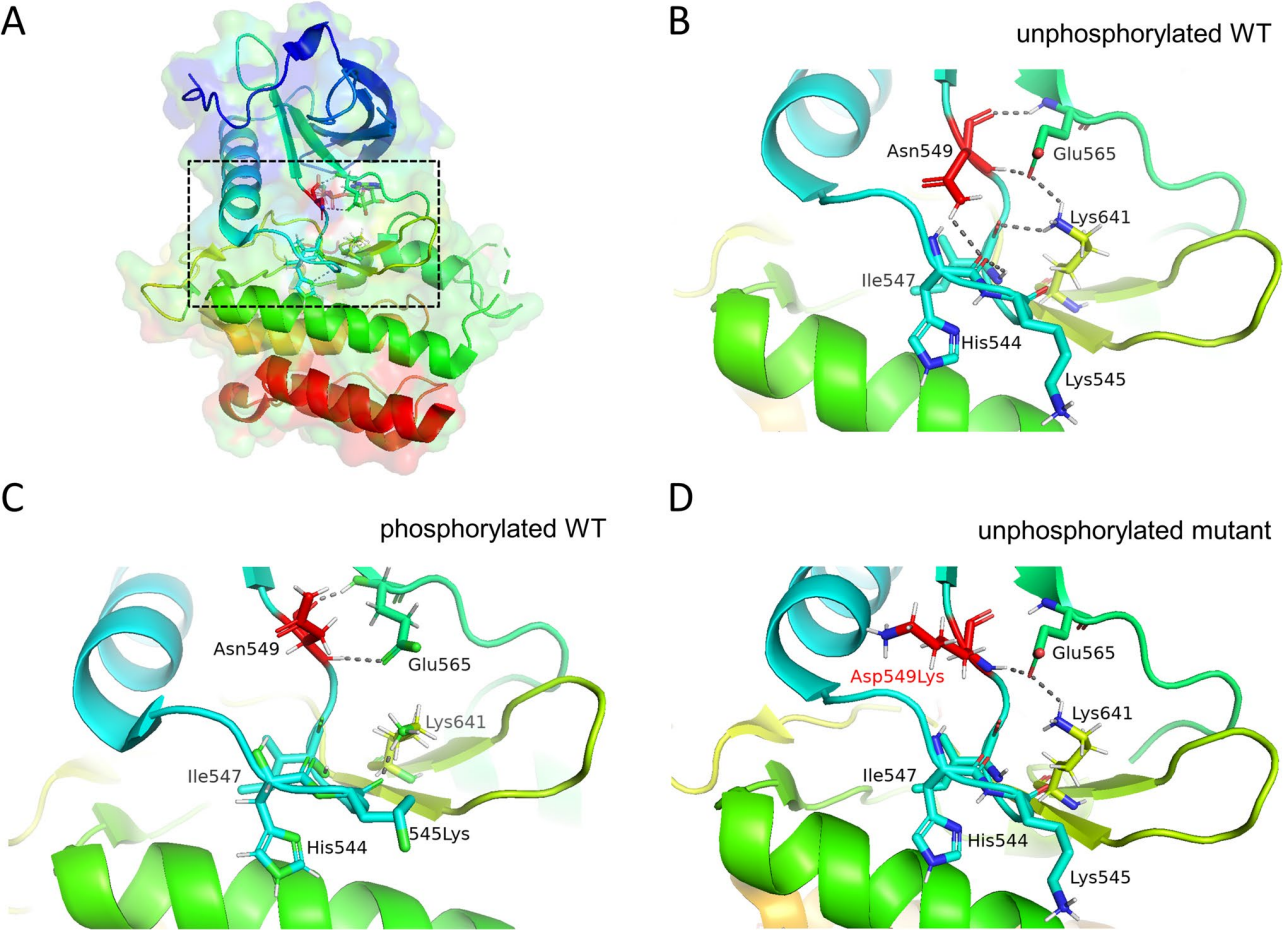
Structure of the FGFR2 kinase domain. **A** Crystal structure of the FGFR2 kinase domain (PDB: 2PVF) shown as cartoon. **B**–**D** The kinase hinge region regulating the kinase activation is shown enlarged, with important residues shown as sticks. **B** In the unphosphorylated wild-type structure of the FGFR2 kinase domain (PDB: 2PSQ) the amino acids Ile547, Asn549, Glu565 and Lys641 form a network of hydrogen bonds which serves as a molecular brake by inhibiting the auto-kinase activity of the FGFR2 receptor (Chen et al. 2007). **C** In the phosphorylated wild-type structure (PDB: 2PVF), this molecular brake is disengaged, resulting in kinase activation (Chen et al. 2007). **D** The variant p.(Asn549Lys) identified in this study compromises the molecular brake in the unphosphorylated state

Structural analysis of the FGFR2 kinase domain revealed that the asparagine at amino acid position 549 is located in the kinase hinge region of FGFR2 (Chen et al. 2007) (Fig. 3A). Therefore, to gain further insight into the potential functional consequences of the p.Asn549Lys variant, we mapped the variant onto the previously determined unphosphorylated (PDB: 2PSQ) and phosphorylated (PDB: 2PVF) structure of the human FGFR2 kinase domain (Chen et al. 2007) (Fig. 4). In the unphosphorylated wild-type structure, the amino acids Ile547, Asn549, Glu565, and Lys641 form a network of hydrogen bonds (Fig. 4B) which serves as a molecular brake by inhibiting the auto-kinase activity of the FGFR2 receptor (Chen et al. 2007). This molecular brake is disengaged in the phosphorylated wild-type structure, resulting in kinase activation (Fig. 4C). Structural mapping shows that substitution of Asn549 by lysine is likely to disrupt the molecular brake even in the unphosphorylated state and thereby induces a ligand-independent kinase activation (Fig. 4D). Structure figures were prepared with PyMol 2.5 (Schrödinger).

### Functional characterization

To characterize the effect of the identified p.(Asn549Lys) variant on FGFR2 function, we generated expression constructs encoding either FLAG-tagged wild-type or mutant FGFR2 and tested their functional outcome by transient overexpression in HEK293T cells. In a first step, we subjected total lysates from cells expressing either wild-type or mutant FGFR2 to western blot analysis and observed similar protein expression levels of wild-type and mutated FGFR2, indicating that the p.(Asn549Lys) variant does not decrease the stability of the mutant FGFR2 protein (Fig. 3C). As the p.(Asn549Lys) variant is located within the tyrosine kinase domain of the receptor, we speculated that this variant might influence the kinase activity of the FGFR2 protein resulting in altered FGFR2 signaling. To analyze the variant’s impact on the tyrosine kinase activity of FGFR2, total lysates from cells expressing FLAG-tagged wild-type or mutant FGFR2 were subjected to immunoprecipitations with anti-FLAG antibodies followed by SDS-PAGE and Western blot analyses with anti-p-Tyr antibodies. We observed that tyrosine phosphorylation in the mutant FGFR2 protein was strongly increased compared to the wild-type FGFR2 (Fig. 3D–E), indicating an increased tyrosine kinase activity of FGFR2 carrying the p.(Asn549Lys) variant.

## Discussion

In this study, we present a girl with cutaneous nevi (widespread nevus sebaceous on the face, skin pigmentation restricted to the right side of her neck, and epidermal nevi along the Blaschko lines) (Fig. 1A–F), malformations of the eye (defects of the retina/choroid, corneal clouding, and strabismus), macrocephaly, and mild developmental delay. Based on the initial clinical tentative diagnosis of SFM syndrome, we extracted genomic DNA from fibroblasts of an affected area in the neck and performed a high-coverage custom-designed multigene panel including *HRAS*, *KRAS*, and *NRAS.* As our intense analysis did not uncover any likely causative variant, we performed ES on DNA extracted from blood and DNA extracted from the skin biopsy. This identified the mosaic *FGFR2* variant NM_000141.5:c.1647=/T > G p.(Asn549=/Lys), and deep sequencing revealed that the variant was present in 32% (45,352/139,782) of the reads from the FFPE sample, in 12% (52,451/450,905) of the fibroblasts and was not detectable in DNA extracted from peripheral blood lymphocytes (Fig. 2A–C).

The fibroblast growth factor receptors comprise a family of related but individually distinct tyrosine kinase receptors. They share a similar structure containing three immunoglobulin-like domains in the extracellular region, a single membrane spanning segment, and a cytoplasmic tyrosine kinase domain (Bochukova et al. 2009). Within this family, FGFR2 is a key regulator in many biological processes, e.g., cell proliferation, tumorigenesis, metastasis, and angiogenesis (Turner and Grose 2010; Azoury et al. 2017; Babina and Turner 2017; Shi et al. 2018). Heterozygous activating non-mosaic germline variants in *FGFR2* have been linked to numerous autosomal dominantly inherited disorders including several craniosynostoses and skeletal dysplasia syndromes such as Apert syndrome (MIM 101200), Crouzon syndrome (MIM 123500), Jackson–Weiss syndrome (MIM 123150), and Pfeiffer syndrome (MIM 101600). In some of the *FGFR2-*associated disorders, the phenotypic spectrum includes skin lesions, such as cutis gyrata in Beare–Stevenson cutis gyrata syndrome (BSTV; MIM 123790) or acne in Apert syndrome (MIM 101200). Interestingly, BSTV syndrome shows besides cutis gyrate additional overlapping phenotypic characteristics including ocular malformations, macrocephaly, and developmental delay. So far, mosaicism, as observed in the reported girl, has not been reported for BSTV syndrome. This might explain why we initially focused on mosaic RASopathies. However, mosaic *FGFR2* alterations have been linked to a number of neoplasms including colon, breast, gastric, endometrial, esophageal and cholangiocarcinoma (Hansen et al. 2005; Kunii et al. 2008; Zhang et al. 2009; Reintjes et al. 2013; Mathur et al. 2014; Kwak et al. 2015; Helsten et al. 2016; Smyth et al. 2017; Shi et al. 2018).

The mosaic missense variant, c.1647T > G, identified in our study is located in exon 12 of the *FGFR2* gene and leads to the substitution of an asparagine at the amino acid position 549 by lysine. This variant has been previously reported as a likely pathogenic somatic variant in an endometrial carcinoma (ClinVar Variation ID: 376156; (Dutt et al. 2008)). Interestingly, structural analysis of the FGFR2 kinase domain revealed that the asparagine at amino acid position 549 is located in the kinase hinge region of FGFR2 (Fig. 4), and it was shown that this region serves as a molecular brake by inhibiting the auto-kinase activity of the receptor (Chen et al. 2007). To characterize the mutational effect of the identified *FGFR2* variant c.1647T > G p.(Asn549Lys), we performed structural mapping, which indicated that substitution of Asn549 by lysine most likely results in a ligand-independent kinase activation by directly disrupting the molecular brake in this region (Fig. 4D). While the constitutive activation of downstream signaling pathways (Fig. 3B) by mosaic activating variants in *HRAS*, *KRAS*, and *NRAS* has been linked to SFM syndrome (Groesser et al. 2012; Lim et al. 2014), *FGFR2* variants have so far not been reported in individuals with this disorder. Our further analysis to explore the functional consequences of the identified postzygotic *FGFR2* variant upstream of the RAS signaling pathway indicated a constitutive tyrosine kinase receptor activation (Fig. 3C–E). Noteworthy, Kuentz et al. reported the mosaic activating *FGFR2* variant NM_000141.4:c.1144=/T > C;p.(Cys382=/Arg) in two fetuses with papillomatous pedunculated sebaceous nevus (PPSN). PPSN, representing a subtype of nevus sebaceous, also typically affects the scalp and face (Kuentz et al. 2017). In contrast to SFM syndrome, however, cerebral, ocular or skeletal anomalies have not been linked as characteristic features to PPSN (Luo et al. 2021). Both described fetuses showed extensive epidermal hyperplasia and were more severely affected than the girl described in our study. In addition, three alternative activating missense variants affecting the same amino acid position as presented here, p.(Asn549His), p.(Asn549Thr) and p.(Asn549Ser), have been described to cause autosomal dominant inherited forms of craniosynostosis (Kan et al. 2002; Wilkie et al. 2007; Apra et al. 2016; Ohishi et al. 2017): the variant p.(Asn549His) has been linked to individuals with the clinical diagnosis of Crouzon syndrome (MIM 123500) (Kan et al. 2002). The variant p.(Asn549Thr) has been identified in cases with typical features of Pfeiffer syndrome (MIM 101600) and Crouzon syndrome (Wilkie et al. 2007; Ohishi et al. 2017). The missense variant p.(Asn549Ser) has been reported in a boy with Crouzon syndrome (Apra et al. 2016). In all three cases, the identified variants were present as heterozygous, non-mosaic germline variants. Chen et al. have shown that the FGFR2 variants p.(Asn549His) and p.(Asn549Thr) lead likewise to a ligand-independent tyrosine kinase activation by directly disengaging the molecular brake (Chen et al. 2007). Furthermore, equivalent substitutions in FGFR3 (p.(Asn540Lys), p.(Asn540Thr) and p.(Asn540Ser)) are known to result in gain of function and to cause hypochondroplasia (MIM 146000), an autosomal dominant skeletal dysplasia characterized by disproportionate short stature (Webster and Donoghue 1997; Deutz-Terlouw et al. 1998; Mortier et al. 2000; Thauvin-Robinet et al. 2003). The causality of the reported variant is also underscored by a striking genotypic and phenotypic overlap with encephalocraniocutaneous lipomatosis (ECCL; MIM 613001), another mosaic neurocutaneous disorder. ECCL is characterized by ocular anomalies, skin lesions (e.g., alopecia, naevus psiloliparus, nodular skin tags, and aplastic scalp defects), and central nervous system anomalies and caused by the postzygotic somatic activating variants p.(Asn546Lys) and p.(Lys656Glu) in FGFR1 (Moog 2009; Bennett et al. 2016). Notably, the FGFR1 variant p.(Asn546Lys) is paralogous to the FGFR2 variant identified in this study. Strikingly, functional studies of ECCL fibroblast cell lines performed by Bennet et al. revealed a constitutive activation of the RAS-MAPK signaling pathway (Bennett et al. 2016). The specific phenotypic features of these various *FGFR*-associated conditions might be explained by multiple factors. First of all, the phenotypic outcome is determined by the level of activation caused by the individual variant. Alternative missense variants might lead to different levels of ligand-independent kinase activation, even if they affect the same amino acid position. Moreover, FGFR1, FGFR2, and FGFR3 have specific expression patterns and diverse tissue distributions during different stages of development (Chen and Deng 2005; Xie et al. 2020). Furthermore, alternative splicing and the distinct isoforms, posttranslational modifications, epigenetic regulation, interaction partners, genetic modifiers, and additional genetic variants contribute to the wide clinical spectrum of *FGFR*-associated diseases (Ornitz et al. 1996; Ornitz and Itoh 2001; Zhang et al. 2006; Zhu et al. 2009; Bao et al. 2021). Besides this, the phenotypic severity of ECCL and the *FGFR2*-associated disorder identified in our study depends in particular on the individual grade of mosaicism and the affected tissues. Taken together, considering the genotypic and phenotypic spectrum of the discussed disorders, *FGFR2*-associated neurocutaneous syndrome seems to be the most accurate clinical-molecular diagnosis for the individual reported here. Furthermore, this delineation comprising both the clinical condition and the molecular etiology is in accordance with the dyadic approach proposed by Biesecker et al. (2021).

The correct molecular diagnosis allows not only accurate genetic counseling but can be also of high relevance with regard to specific surveillance and management strategies. As the variant p.(Asn549Lys) in FGFR2 has previously been linked to an endometrial endometrioid adenocarcinoma (Dutt et al. 2008), we hypothesize that the presented mosaic *FGFR2*-associated neurocutaneous syndrome is associated with an increased tumor risk, although further cases are needed to verify this. Notably, ECCL is also associated with increased risk of developing different kinds of cancer and the molecular confirmation of suspected ECCL enables specific treatment options (Moog and Dobyns 1993; Bennett et al. 2016; Valera et al. 2018; Barry et al. 2023). In line with this, there are clinical trials ongoing for patients with FGFR-altered carcinoma to investigate FGFR kinase inhibitors as treatment options (Krook et al. 2020; Jaidee et al. 2022). Intriguingly, the high relevance of a correct molecular diagnosis as the basis for developing novel treatment options has already been demonstrated for other mosaic syndromes. For children with Proteus syndrome, for example, Miransertib, an orally available, highly selective pan-AKT inhibitor initially developed for cancer therapeutics, can be a potential therapeutic option (Keppler-Noreuil et al. 2019; Forde et al. 2021).

In summary, the identification of a mosaic activating *FGFR2* variant in an individual with a specific neurocutaneous phenotype and the initial clinical diagnosis of SFM syndrome highlights a tremendous overlap of the genotypic and phenotypic spectrum of mosaic RASopathies and *FGFR*-associated syndromes. The reported findings demonstrate the challenges in dealing with those ultra-rare mosaic conditions. Molecular analysis of *FGFR2* should especially be considered in the genetic workup of individuals with the suspected diagnosis of a mosaic neurocutaneous condition such as BSTV syndrome, ECCL, or SFM syndrome, as the knowledge of the molecular cause might have relevant implications for genetic counseling, prognosis, tumor surveillance, and potential treatment options.

## Data Availability

The data that support the findings of this study are available on request from the corresponding author. The data are not publicly available due to privacy or ethical restrictions.
